# Interleukin-6 stimulates gene expression of extracellular matrix components in bovine mesangial cells in culture

**DOI:** 10.1155/S0962935193000614

**Published:** 1993

**Authors:** C. Zoja, A. Benigni, G. Piccinini, M. Figliuzzi, L. Longaretti, G. Remuzzi

**Affiliations:** 1 Mario Negri Institute for Pharmacological Research via Gavazzeni 11 Bergamo Italy; 2 Division of Nephrology Ospedali Riuniti di Bergamo Bergamo 24125 Italy

## Abstract

The effect of interleukin-6 (IL-6) on gene expression of extracellular matrix components in bovine mesangial cells in culture has been investigated. IL-6 (100 U/ml) time dependently increased the steady state expression of mRNAs coding for α1 collagen III and fibronectin, both transcripts being 1.5- and 2.5-fold higher than basal level at 24 and 48 h, respectively. In contrast, IL-6 stimulated laminin mRNA expression only after 48 h incubation (2.5-fold upon basal level). These results suggest that IL-6 could favour glomerular matrix accumulation thus contributing to the development of glomerulosclerosis.


Mediators of Inflammation 2, 429–433 (1993)

### Interleukin-6 stimulates gene expression of extracellular matrix components in bovine mesangial cells in culture

C. Zoja,[1] A. Benigni,[1] G. Piccinini,[1] M. Figliuzzi,[1] L. Longaretti[1] and G. Remuzzi[1,2,CA]

[1]Mario Negri Institute for Pharmacological Research, via Gavazzeni 11 and [2]Division of Nephrology, Ospedali Riuniti di Bergamo, 24125 Bergamo, Italy

[CA] Corresponding Author

**THE effect of interleukin-6 (IL-6) on gene expression of extracellular matrix components in bovine mesangial cells in culture has been investigated. IL-6 (100 U/ml) time dependently increased the steady state expression of mRNAs coding for α1 collagen III and fibronectin, both transcripts being 1.5- and 2.5-fold higher than basal level at 24 and 48 h, respectively. In contrast, IL-6 stimulated laminin mRNA expression only after 48 h incubation (2.5-fold upon basal level). These results suggest that IL-6 could favour glomerular matrix accumulation thus contributing to the development of glomerulosclerosis.**

**Key words:** Extracellular matrix, Gene expression, Interleukin-6, Mesangial cells

### Introduction

Accumulation of extracellular matrix and proliferation of intrinsic glomerular cells are abnormalities common to several forms of glomerular diseases which might contribute to renal disease progression.[1,2] The generation of extracellular matrix is at least, in part, regulated by cytokines and growth factors produced by infiltrating or resident cells in the glomerulus. Thus transforming growth factor $\beta$ (TGF$\beta$) and platelet-derived growth factor (PDGF) have been recognized as possible modulators of matrix protein synthesis by glomerular cells contributing to increased matrix formation in experimental glomerulonephritis.[3–7]

Among other cytokines, interleukin-6 (IL-6) has very interesting potential as a new mediator of glomerular injury and disease progression. IL-6 is a pleiotropic cytokine that acts on a wide range of tissues, exerting growth-inducing, growth-inhibitory and differentiation-inducing effects, depending on the nature of the target cells.[8] Originally described as a product of activated monocytes and lymphocytes,[8] IL-6 is synthesized by many cell types including glomerular mesangial cells.

The authors have recently shown that human mesangial cells in culture stimulated with interleukin-1 (IL-1) and tumour necrosis factor, unlike resting mesangial cells, express IL-6 gene and release the corresponding protein in the supernatant.[9] Other authors have reported the capability of unstimulated rat mesangial cells to secrete and to proliferate in response to exogenously added IL-6.[10] Thus a role of IL-6 as an autocrine growth factor for mesangial cells was proposed.[10] In keeping with this possibility are recent clinical data showing an increased urinary excretion of IL-6 in patients with mesangial proliferative glomerulonephritis.[11] Moreover experimental studies have documented that IL-6 transgenic mice, carrying a human IL-6 genomic gene fused with human immunoglobulin heavy chain enhancer, had high serum IL-6, proteinuria, and mesangial proliferative glomerulonephritis[12] and that treatment with anti-human IL-6 antibody prevented mesangial cell proliferation.[13] In the experimental model of lupus nephritis in mice it has been shown that the administration of recombinant IL-6 accelerated the development of the autoimmune glomerulonephritis.[14] However, whether IL-6 has indeed mitogenic properties on mesangial cells is still a very controversial issue considering that in other recent experiments IL-6 inhibits rather than stimulates the growth of mesangial cells in culture.[15] The *in vivo* finding that pretreatment with IL-6 reduces albumin excretion in a rat model of nephrotoxic nephritis[16] is consistent with the possibility that IL-6, by inhibiting mesangial growth, protects against glomerular damage. Since cultured mesangial cells constitutively express the 80 kDa IL-6 receptor and the IL-6 signal transducer, gp 130,[17] the authors explored whether IL-6 regulated gene expression of extracellular matrix components in bovine mesangial cells in culture.

### Methods

*Mesangial cell cultures:* Mesangial cells were obtained from collagenase treated isolated bovine glomeruli

© 1993 Rapid Communications of Oxford Ltd as described previously.[18] Cells were cultured in RPMI 1640 medium (Gibco, Grand Island, NY) supplemented with 20 mM Hepes (Sigma Chemical Company, St. Louis, MO), 2 mM glutamine (Gibco), 100 units/ml penicillin, 100 μg/ml of streptomycin, 250 ng/ml of fungizone and 20% foetal calf serum (Gibco). Confluent cells were passed by washing with $Ca^{2+}$-free, $Mg^{2+}$-free Hank's balanced solution followed by incubation with 0.05% trypsin/0.02% ethylenediaminetetraacetic acid and resuspension in complete RPMI 1640 medium. Cells were used between passages 10 to 13. Cells were identified by phase contrast microscopy and by staining for intermediate filaments as described previously[18] (Fig. 1A and B).

*Experimental design:* Confluent bovine mesangial cells grown in 100 mm plastic dishes were kept under serum-free conditions for 48 h. Cells deprived of serum were incubated in the presence or absence of human recombinant IL-6 (100 U/ml; provided by Dr S. Gillis, Immunex, Seattle, WA) for different time intervals. At the end of the incubation, the cells were used for total cellular RNA preparation in order to study α1 collagen III, fibronectin and laminin gene expression.

*Preparation of total cellular RNA and Northern blot analysis:* Total cellular RNA was isolated from bovine mesangial cells as described previously[19] by lysing cells in guanidium isothiocyanate and recovering RNA by centrifugation through caesium chloride. Seven-microgram samples were then fractionated on a 0.7% agarose gel with 6% formaldehyde and blotted onto synthetic membranes (Gene Screen Plus, New England Nuclear, Boston, MA). All gels were stained with ethidium bromide to visualize 28S and 18S ribosomal RNA bands. These bands were used to confirm that (a) approximately equivalent amounts of RNA were loaded in each gel lane, and (b) there was no obvious degradation of RNA. cDNA probes for human α1 collagen III (gift of Dr R. Nischt, Dermatologische Klinik der Universitat, Koln, Germany), human fibronectin (purchased from HGMP Resource Centre, Harrow, Middx, UK) and mouse laminin (gift of Dr I. Oberbaumer, Max Planck Institut fur Biochemie, Munich, Germany) were labelled to a specific activity of $10^9$ cpm/μg by using hexanucleotide primers and $^{32}$P-dCTP.[20]

FIG. 1. Phase contrast micrograph of bovine mesangial cells in culture stained by indirect immunofluorescence for the intermediate filaments desmin (A) or vimentin (B).

Hybridization was performed for 20 h at 60°C in a solution containing 1 M NaCl, 1% sodium dodecyl sulfate (SDS), 10% dextran sulfate, 100 μg/ml salmon sperm DNA, and 1 × $10^6$ cpm/ml labelled probe as described previously.[9] The membranes were washed with 1 × standard saline citrate (SSC)/1% SDS for 1 h at 60°C and 0.1 × SSC at room temperature for 1 h (1 × SSC = 0.15 M NaCl and 0.015 M Na citrate, pH 7.0). The blots were then dried and used to expose Kodak Xomat X-ray film with intensifying screens. Membranes were subsequently rehybridized with rat GAPDH cDNA as 'housekeeping gene' to determine an internal standard of total RNA content. After optimal exposure, the autoradiographs of each experiment were scanned by a laser densitometer in order to quantify the relative amounts of radioactively labelled probe bound for each transcript. α1 collagen III, fibronectin and laminin mRNA optical density was normalized to that of the constituently released GAPDH gene expression.

### Results

In a first series of experiments the effect of IL-6 on the steady state level of α1 collagen III specific mRNA in bovine mesangial cells after 3, 6, 24 and 48 h incubation (Fig. 2) was measured. Densitometric analysis of the autoradiographic signals showed that in unstimulated mesangial cells collagen type III transcript levels were comparable during all the observation time. In mesangial cells stimulated with IL-6 transcriptional rates of collagen III gene were comparable with those of resting cells after 3 and 6 h incubation. An increase in collagen III gene expression was observed after 24 and 48 h of IL-6 stimulation, the mRNA levels

FIG. 2. Northern blot analysis of α1 collagen III mRNA from cultured bovine mesangial cells (MC) exposed for 3, 6, 24 and 48 h to medium alone or IL-6 (100 U/ml). Blots are representative of three individual experiments. The same membrane was rehybridized to the GAPDH probe. Inset: ethidium bromide stain of RNA.

being 1.5- and 2.5-fold higher than corresponding unstimulated control cells, respectively. Similar results were obtained in a next series of experiments when the effect of IL-6 on fibronectin mRNA was tested (Fig. 3). Starting 24 h after addition of IL-6 a 1.5-fold increase in fibronectin specific mRNA levels was observed. A maximal increase (2.5-fold over unstimulated control cells) was seen after 48 h. In untreated mesangial cells the level of fibronectin specific mRNA did not change with time. Finally, we investigated whether IL-6 also stimulated laminin mRNA in bovine mesangial cells. Figure 4 shows that IL-6 did not change laminin mRNA levels in mesangial cells after 6 and 24 h incubation.

FIG. 3. Fibronectin mRNA expression in cultured bovine mesangial cells (MC). Data shown are from a representative experiment ($n = 3$). Total cellular RNA was isolated after incubation of cells with medium alone or IL-6 (100 U/ml) for different time intervals. The same membrane was rehybridized to the GAPDH probe. Inset: ethidium bromide stain of RNA.

FIG. 4. Laminin mRNA expression in cultured bovine mesangial cells (MC) incubated for 6, 24 and 48 h with medium alone or IL-6 (100 U/ml). Data shown are from a representative experiment ($n = 3$). The same membrane was rehybridized to the GAPDH probe. Inset: ethidium bromide stain of RNA.

IL-6 increased laminin mRNA expression only after 48 h incubation. At this time densitometric analysis revealed a 2.5-fold increase of laminin transcripts in IL-6 stimulated cells compared with control cells.

### Discussion

The present data show that IL-6 time dependently increases gene expression of extracellular matrix components in bovine mesangial cells and indicate that IL-6 could directly affect accumulation of glomerular extracellular matrix. The stimulation of the extracellular matrix by IL-6 has been reported previously for cell types other than mesangial cells. Lanser and Brown[21] showed that IL-6 directly stimulated fibronectin production by rat hepatocytes in a dose-dependent manner.

Increased deposition of extracellular matrix components within the glomerulus is considered a major determinant of glomerulosclerosis.[2] The process of glomerular accumulation of the extracellular matrix may be affected by a variety of factors that are active either at the level of extracellular matrix generation or degradation. Experimental studies have greatly contributed to the clarification of the role of cytokines and growth factors in the modulation of the amount of deposited extracellular matrix. Thus polypeptide mediators like TGF$\beta$, PDGF and IL-1 have been shown to stimulate both extracellular matrix protein production or degradation through the activation of proteinases.[22–24] In some instances, also the distribution of matrix proteins can be altered during glomerular disease. Immunohistochemical and biochemical studies have demonstrated that in normal conditions major components of mesangial matrix include collagen IV, fibronectin, laminin, entactin/nidogen and proteoglycans. In diseased glomeruli also the insterstitial collagens I and III have been localized.[25,26] These collagens are scarce or undetectable in normal glomeruli of laboratory animals and humans. However studies with cultured mesangial cells have shown that mRNA of collagens I and III are expressed and translated into secreted proteins.[23,27] Thus, it is likely that in glomerular diseases mesangial cells are a source of collagens and other extracellular matrix proteins that accumulate, resulting in mesangial and glomerular scarring. The finding that IL-6 enhances gene expression of extracellular matrix proteins in mesangial cells suggests that this cytokine by promoting extracellular matrix deposition could play a role in the processes leading to glomerulosclerosis.

### References

1. Klahr S, Schreiner G, Ichikawa I. The progression of renal disease. *New Engl J Med* 1988; **318**: 1657–1666.
2. Bruijn JA, Hogendoron PCW, Hoedemaker PJ, Fleuren GJ. The extracellular matrix in pathology. *J Lab Clin Med* 1988; **111**: 140–149.
3. Border W, Okuda S, Languino LR, Ruoslahti E. Transforming growth factor $\beta$ regulates production of proteoglycans by mesangial cells. *Kidney Int* 1990; **37**: 689–695.
4. Nakamura T, Miller D. Ruoslahti E, Border W. Production of extracellular matrix by glomerular cells is regulated by transforming growth factor $\beta$1. *Kidney Int* 1992; **41**: 1221–1231.
5. Okuda S, Languino LR, Ruoslahti E, Border W. Elevated expression of transforming growth factor $\beta$ and proteoglycan production in experimental glomerulonephritis. *J Clin Invest* 1990; **86**: 453–462.
6. Suzuki S, Ebihara I, Nakamura T, Tomino Y, Koide H. Effects of peptide growth factors on regulation of extracellular matrix gene expression in cultured rat mesangial cells. *J Am Soc Nephrol* 1991; **2**: 446 (abstract).
7. Johnson RJ, Raines EW, Floege J, *et al.* Inhibition of mesangial cell proliferation and matrix expression in glomerulonephritis in the rat by antibody to platelet-derived growth factor. *J Exp Med* 1992; **175**: 1413–1416.
8. Hirano T, Akira S, Taga T, Kishimoto T. Biological and clinical aspects of interleukin 6. *Immunol Today* 1990; **11**: 443–449.
9. Zoja C, Wang JM, Bettoni S, *et al.* Interleukin-1$\beta$ and tumor necrosis factor-$\alpha$ induce gene expression and production of leukocyte chemotactic factors, colony-stimulating factors, and interleukin-6 in human mesangial cells. *Am J Pathol* 1991; **138**: 991–1003.
10. Ruef C, Budde K, Lacy J, *et al.* Interleukin 6 is an autocrine growth factor for mesangial cells. *Kidney Int* 1990; **38**: 249–257.
11. Horii Y, Muraguchi A, Iwano M, *et al.* Involvement of IL-6 in mesangial proliferative glomerulonephritis. *J Immunol* 1989; **143**: 3949–3955.
12. Suematsu S, Matsuda T, Aozasa K, *et al.* IgG1 plasmacytosis in interleukin 6 transgenic mice. *Proc Natl Acad Sci USA* 1989; **86**: 7547–7551.
13. Matsusaka T, Suematsu S, Horii Y, *et al.* Generation of mesangial proliferative glomerulonephritis in interleukin-6 transgenic mice. (Abstract). *Proceedings XIth International Congress of Nephrology*, Tokyo, Japan, 15–20 July, 1990, p. 374A.
14. Car B, Eugster HP, Weber M, Ryffel B. Interleukin-6 enhances glomerulonephritis in (NZBxW)F1 mice. (Abstract). *Proceedings XIIth International Congress of Nephrology*, Jerusalem, Israel, 13–18 June, 1993, p. 66.
15. Ikeda M, Ikeda U, Ohara T, Kusano E, Kano S. Recombinant interleukin-6 inhibits the growth of rat mesangial cells in culture. *Am J Pathol* 1992; **141**: 327–334.
16. Karkar AM, Tam FWK, Proudfoot A, Rees AJ. Modulation of glomerular injury in nephrotoxic nephritis by hrIL-6. *J Am Soc Nephrol* 1992; **3**: 596 (abstract).
17. Rambaldi A, Bettoni S, Remuzzi G, Zoja C. Human mesangial cells in culture express interleukin-6 receptor and interleukin-6 signal transducer, gp 130. *J Am Soc Nephrol* 1992; **3**: 612 (abstract).
18. Zoja C, Benigni A, Renzi D, Piccinelli A, Perico N, Remuzzi G. Endothelin and eicosanoid synthesis in cultured mesangial cells. *Kidney Int* 1990; **37**: 927–933.
19. Rambaldi A, Young DC, Griffin JD. Expression of the M-CSF (CSF-1) gene by human monocytes. *Blood* 1987; **69**: 1409–1413.
20. Feinberg AP, Vogelstein B. A technique for radiolabelling DNA restriction endonuclease fragments to high specific activity. *Anal Biochem* 1983; **132**: 6.
21. Lanser ME, Brown GE. Stimulation of rat hepatocyte fibronectin production by monocyte-conditioned medium is due to interleukin 6. *J Exp Med* 1989; **170**: 1781–1786.
22. Roberts AB, McCune BK, Sporn MB. TGF-$\beta$: Regulation of extracellular matrix. *Kidney Int* 1992; **41**: 557–559.
23. Sterzel RB, Schulze-Lohoff E, Weber M, Goodman SL. Interactions between glomerular mesangial cells, cytokines, and extracellular matrix. *J Am Soc Nephrol* 1992; **2**: S126–S131.
24. Sedor JR. Cytokines and growth factors in renal injury. *Semin Nephrol* 1992; **12**: 428–440.
25. Morel-Maroger Striker L, Killen PD, Chi E, Striker GE. The composition of glomerulosclerosis. 1. Studies in focal sclerosis, crescentic glomerulonephritis, and membranoproliferative glomerulonephritis. *Lab Invest* 1984; **51**: 181–192.
26. Oomura A, Nakamura T, Arawa M, Ooshima A, Isemura M. Alterations in the extracellular matrix components in human glomerular diseases. *Virchows Archiv A Pathol Anat* 1989; **415**: 151–159.
27. Ishimura E, Sterzel RB, Budde K, Kashgarian M. Formation of extracellular matrix by cultured rat mesangial cells. *Am J Pathol* 1989; **134**: 843–855.

ACKNOWLEDGEMENT. This paper was supported in part by the CNR (National Research Council, Rome, Italy), contract no. 93. 01734. CT04.

**Received 26 July 1993; accepted in revised form 20 September 1993**

## References

[PDF_00317] Border W. A., Okuda S., Languino L. R., Ruoslahti E. (1990). Transforming growth factor-beta regulates production of proteoglycans by mesangial cells.. Kidney Int.

[PDF_00315] Bruijn J. A., Hogendoorn P. C., Hoedemaeker P. J., Fleuren G. J. (1988). The extracellular matrix in pathology.. J Lab Clin Med.

[PDF_00365] Feinberg A. P., Vogelstein B. (1983). A technique for radiolabeling DNA restriction endonuclease fragments to high specific activity.. Anal Biochem.

[PDF_00332] Hirano T., Akira S., Taga T., Kishimoto T. (1990). Biological and clinical aspects of interleukin 6.. Immunol Today.

[PDF_00384] Ishimura E., Sterzel R. B., Budde K., Kashgarian M. (1989). Formation of extracellular matrix by cultured rat mesangial cells.. Am J Pathol.

[PDF_00329] Johnson R. J., Raines E. W., Floege J., Yoshimura A., Pritzl P., Alpers C., Ross R. (1992). Inhibition of mesangial cell proliferation and matrix expansion in glomerulonephritis in the rat by antibody to platelet-derived growth factor.. J Exp Med.

[PDF_00313] Klahr S., Schreiner G., Ichikawa I. (1988). The progression of renal disease.. N Engl J Med.

[PDF_00367] Lanser M. E., Brown G. E. (1989). Stimulation of rat hepatocyte fibronectin production by monocyte-conditioned medium is due to interleukin 6.. J Exp Med.

[PDF_00375] Morel-Maroger Striker L., Killen P. D., Chi E., Striker G. E. (1984). The composition of glomerulosclerosis. I. Studies in focal sclerosis, crescentic glomerulonephritis, and membranoproliferative glomerulonephritis.. Lab Invest.

[PDF_00320] Nakamura T., Miller D., Ruoslahti E., Border W. A. (1992). Production of extracellular matrix by glomerular epithelial cells is regulated by transforming growth factor-beta 1.. Kidney Int.

[PDF_00323] Okuda S., Languino L. R., Ruoslahti E., Border W. A. (1990). Elevated expression of transforming growth factor-beta and proteoglycan production in experimental glomerulonephritis. Possible role in expansion of the mesangial extracellular matrix.. J Clin Invest.

[PDF_00381] Oomura A., Nakamura T., Arakawa M., Ooshima A., Isemura M. (1989). Alterations in the extracellular matrix components in human glomerular diseases.. Virchows Arch A Pathol Anat Histopathol.

[PDF_00363] Rambaldi A., Young D. C., Griffin J. D. (1987). Expression of the M-CSF (CSF-1) gene by human monocytes.. Blood.

[PDF_00370] Roberts A. B., McCune B. K., Sporn M. B. (1992). TGF-beta: regulation of extracellular matrix.. Kidney Int.

[PDF_00338] Ruef C., Budde K., Lacy J., Northemann W., Baumann M., Sterzel R. B., Coleman D. L. (1990). Interleukin 6 is an autocrine growth factor for mesangial cells.. Kidney Int.

[PDF_00372] Sterzel R. B., Schulze-Lohoff E., Weber M., Goodman S. L. (1992). Interactions between glomerular mesangial cells, cytokines, and extracellular matrix.. J Am Soc Nephrol.

[PDF_00340] Suematsu S., Matsuda T., Aozasa K., Akira S., Nakano N., Ohno S., Miyazaki J., Yamamura K., Hirano T., Kishimoto T. (1989). IgG1 plasmacytosis in interleukin 6 transgenic mice.. Proc Natl Acad Sci U S A.

[PDF_00360] Zoja C., Benigni A., Renzi D., Piccinelli A., Perico N., Remuzzi G. (1990). Endothelin and eicosanoid synthesis in cultured mesangial cells.. Kidney Int.

[PDF_00334] Zoja C., Wang J. M., Bettoni S., Sironi M., Renzi D., Chiaffarino F., Abboud H. E., Van Damme J., Mantovani A., Remuzzi G. (1991). Interleukin-1 beta and tumor necrosis factor-alpha induce gene expression and production of leukocyte chemotactic factors, colony-stimulating factors, and interleukin-6 in human mesangial cells.. Am J Pathol.

